# Identification and validation of a regulatory mutation upstream of the *BMP2* gene associated with carcass length in pigs

**DOI:** 10.1186/s12711-021-00689-0

**Published:** 2021-12-14

**Authors:** Jing Li, Song Peng, Liepeng Zhong, Lisheng Zhou, Guorong Yan, Shijun Xiao, Junwu Ma, Lusheng Huang

**Affiliations:** National Key Laboratory for Swine Genetics, Breeding and Production Technology, Jiangxi Agricultural University, Nanchang, 330045 China

## Abstract

**Background:**

Carcass length is very important for body size and meat production for swine, thus understanding the genetic mechanisms that underly this trait is of great significance in genetic improvement programs for pigs. Although many quantitative trait loci (QTL) have been detected in pigs, very few have been fine-mapped to the level of the causal mutations. The aim of this study was to identify potential causal single nucleotide polymorphisms (SNPs) for carcass length by integrating a genome-wide association study (GWAS) and functional assays.

**Results:**

Here, we present a GWAS in a commercial Duroc × (Landrace × Yorkshire) (DLY) population that reveals a prominent association signal (*P* = 4.49E−07) on pig chromosome 17 for carcass length, which was further validated in two other DLY populations. Within the detected 1 Mb region, the *BMP2* gene stood out as the most likely causal candidate because of its functions in bone growth and development. Whole-genome gene expression studies showed that the *BMP2* gene was differentially expressed in the cartilage tissues of pigs with extreme carcass length. Then, we genotyped an additional 267 SNPs in 500 selected DLY pigs, followed by further whole-genome SNP imputation, combined with deep genome resequencing data on multiple pig breeds. Reassociation analyses using genotyped and imputed SNP data revealed that the rs320706814 SNP, located approximately 123 kb upstream of the *BMP2* gene, was the strongest candidate causal mutation, with a large association with carcass length, with a ~ 4.2 cm difference in length across all three DLY populations (N = 1501; *P* = 3.66E−29). This SNP segregated in all parental lines of the DLY (Duroc, Large White and Landrace) and was also associated with a significant effect on body length in 299 pure Yorkshire pigs (*P* = 9.2E−4), which indicates that it has a major value for commercial breeding. Functional assays showed that this SNP is likely located within an enhancer and may affect the binding affinity of transcription factors, thereby regulating *BMP2* gene expression.

**Conclusions:**

Taken together, these results suggest that the rs320706814 SNP on pig chromosome 17 is a putative causal mutation for carcass length in the widely used DLY pigs and has great value in breeding for body size in pigs.

**Supplementary Information:**

The online version contains supplementary material available at 10.1186/s12711-021-00689-0.

## Background

Body size, usually measured as body height or length, is not only an important indicator of human growth and health [1] but is also closely related with growth rate, carcass yield, and carcass composition in livestock [2], and is therefore an economically important trait for livestock. Body or carcass length (CL) of pigs varies considerably between pig breeds [3]. Due to its moderate to high heritability and significant phenotypic and genetic relationships with other economic traits, CL has been one of the major objectives of pig breeding programs [4–6].

To uncover the genetic architecture of CL in pigs, quantitative trait loci (QTL) for this trait have been detected by linkage mapping or genome-wide association studies (GWAS), usually using an F2 cross between outbred lines, such as a European commercial breed with wild boar or with a local native breed or another commercial breed [7–13]. GWAS enables the identification of QTL in populations of less related individuals. Recently, QTL for carcass traits have been identified by GWAS in several purebred and commercial crossbred populations [14–17]. However, to our knowledge, few studies have attempted to detect QTL for carcass traits in commercial Duroc × Landrace × Yorkshire (DLY) hybrid pigs, which account for the largest proportion of pork production in China (> 80%) and even in the world. China produces and slaughters nearly 700 million DLY pigs per year. Therefore, it is of great economic value to increase the CL and productive performance of DLY pigs.

Many GWAS in pigs have used the Illumina porcine SNP60 BeadChip with 62,163 single nucleotide polymorphisms (SNPs) to genotype animals. In general, trait-associated SNPs identified by GWAS are in strong linkage disequilibrium (LD) with the causal mutation in the studied population, but this may not be the case in other populations with the same QTL because different populations can have different LD patterns. Therefore, for practical applications in breeding, it is important to identify the causal gene and causal mutation for a QTL that has a strong effect on an economic trait.

Many QTL have been detected in pigs but very few have been fine-mapped to the level of the causal mutation [18]. The resolution of QTL mapping is affected by many factors, such as phenotyping accuracy, the LD structure of the population, sample size, marker density, and the statistical methods used. As whole-genome sequencing (WGS) data are superior to the current SNP chips in terms of marker density and are expected to cover most of the causal SNPs, association studies using WGS data are becoming more widespread in human and animal genetics research [19–21]. Of course, although the costs of WGS are decreasing, it is still relatively expensive to sequence a large number of animals. A cost-effective approach is to impute from lower density SNP chips to WGS for the target population based on the WGS data of some sequenced individuals [22].

Fine-mapping is a statistical analysis approach that is designed to assign probabilities of causality to candidate variants located in regions identified by GWAS. Recently, several Bayesian methods, including CAVIARBF, CAVIAR, and PAINTOR, have been developed to incorporate GWAS summary statistics into fine-mapping analyses and to calculate posterior probabilities of causality for SNPs across all loci of interest [23–25].

In this study, we identified a unique significant QTL for CL in DLY pigs by 60 K GWAS and then fine-mapped the causal variant through both regional association analysis in multiple populations and Bayesian analysis with information on genotyped and imputed SNPs in the QTL region. We also conducted functional validation and molecular biology studies to determine the causal SNP for this QTL.

## Methods

### Populations and phenotype

This study involved three crossbred DLY and a purebred Yorkshire pig population. As far as we know, the four populations are not related to each other. The three DLY populations (denoted DLY-P1, DLY-P2 and DLY-P3) comprised 686, 608, and 207 pigs, respectively, were raised by different farm enterprises, and slaughtered at approximately 180 days of age in the same commercial abattoir in Nanchang city in 2011, 2012, and 2015, respectively. Carcass length (CL) was measured from the first cervical vertebra to the pubis on all pigs, while in the DLY-P3, the length of the tenth thoracic vertebra was also measured. For the Yorkshire population, body length was measured on 299 pigs at approximately 5 months of age on a farm owned by Fujian Yichun Agricultural Development Co., Ltd.

### Genotyping and quality control

Genomic DNA was isolated from ear tissue using a standard phenol/chloroform extraction method and was adjusted to a concentration of 50 to 80 ng/µL using a Nanodrop-1000 spectrophotometer (Thermo Fisher, USA). All pigs from the DLY-P1 population were genotyped with the Illumina PorcineSNP60 BeadChip for the purpose of GWAS analysis. Quality control was conducted using the PLINK v1.07 software [26]. SNPs with a call rate lower than 90% or a minor allele frequency (MAF) lower than 5% were eliminated. Individuals with an overall call rate lower than 95% were removed. After filtering, 46,351 SNPs remained for further statistical analyses.

### GWAS analysis

Genome-wide association analyses of body size were conducted using the GenABEL package in the R software [27]. Associations between CL and SNPs were analyzed using the following generalized linear mixed model [28]:

$$\mathbf{y}=\mathbf{1}\mu +\mathbf{X}\mathbf{b}+\mathbf{S}\mathrm{c}+\mathbf{Z}\mathbf{a}+\mathbf{e},$$

where $\mathbf{y}$ is a vector of phenotypes; $\mu$ is the overall mean; $\mathbf{b}$ is a vector of fixed effects including sex and batch; $\mathrm{c}$ is the allele substitution effect for the fitted SNP (one at a time); $\mathbf{a}$ is a vector of random additive genetic effects, assumed to follow a multinomial distribution $\mathbf{a}\sim N(\mathbf{0}, \mathbf{G}{\upsigma }_{\alpha }^{2})$, where $\mathbf{G}$ is the genomic relationship matrix and ${\upsigma }_{\alpha }^{2}$ is the polygenetic additive variance; $\mathbf{e}$ is a vector of residual errors with $\mathbf{e}\sim N(\mathbf{0}, \mathbf{I}{\upsigma }_{\mathrm{e}}^{2})$, where $\mathbf{I}$ is the identity matrix and ${\upsigma }_{\mathrm{e}}^{2}$ is the residual variance; and $\mathbf{X}$, $\mathbf{S}$, and $\mathbf{Z}$ are the incidence matrices for $\mathbf{b}$, $\mathrm{c}$, and **a**, respectively. The suggestive (*P* = 2.16E−05) and genome-wide (*P* = 1.07E−06) significance thresholds were determined by the Bonferroni method [29]. The phenotypic variance explained by each top GWAS SNP was calculated as (V_reduce_−V_full_)/V_reduce_, where V_full_ and V_reduce_ are estimates of the residual variance of ordinary linear models with and without SNP terms, respectively.

Population stratification was assessed by examining the distribution of test statistics and assessing their deviation from the null distribution (i.e., the distribution expected under the null hypothesis that the fitted SNP is not associated with the trait) in a quantile–quantile (Q-Q) plot [30]. The Q-Q plots were constructed using the R software. Linkage disequilibrium (LD) between tested SNPs was estimated by using the Haploview version 4.2 software [31]. For the DLY-P1 population, the LD decay pattern was analyzed using the PopLD decay software [32]. SNP-based haplotypes in the QTL interval were constructed using the BEAGLE software [33], and the effects of different haplotypes on CL were compared by analysis of variance using a general linear model.

### Genotype imputation and regional association analysis

Based on the SNP annotation information from the *Sus scrofa* reference genome sequence (Sscrofa11.1) and the SNP dataset from the whole-genome resequencing data of western commercial pigs, we selected 267 SNPs that were nearly evenly distributed across a QTL region on *Sus scrofa* (SSC) chromosome 17 that was identified by GWAS and segregated in at least one of the three commercial lines (Duroc, Landrace or Yorkshire). To save on costs and improve the power of QTL detection, we also selected 200 (top 100 and bottom 100) and 300 individuals (top 150 and bottom 150) with extreme CL phenotypes from DLY-P1 and DLY-P2, respectively, to form the DLY-Pf population for QTL fine-mapping analysis. Then, the 267 SNPs were genotyped in the DLY-Pf using the SNP scan technology based on double ligation and multiplex fluorescence PCR (Genesky Biotech, Co., Ltd, Shanghai, China). Potential population stratification was assessed by principal component analysis (PCA) using the PLINK software. Then, imputation from the genotyped SNPs to whole-sequence for the QTL region on SSC17 was performed on the mixed population using the Beagle 4.1 software with default parameter settings. Ultimately, we obtained genotype data for 11,029 SNPs in this region, of which, 9149 passed the quality control criteria mentioned previously and were included in the subsequent regional association analysis.

### Bayesian fine-mapping association

The Bayesian method provides a powerful way to refine association signals or identify potential causal SNPs in GWAS-detected regions [34]. We used the CAVIARBF software to perform Bayesian analysis [23]. For quantitative traits, the inputs were the marginal test statistics for each SNP, which is the t-statistic from the linear regression model. Using this framework, the posterior probability for each SNP can be calculated. We created credible sets that ranked associations in descending order of posterior probability. We considered a posterior probability greater than 0.95 as indicative of a potential causal mutation.

### Complete sequencing of the *BMP2* gene

The *bone morphogenetic protein 2* (*BMP2*) gene is a strong candidate gene for the QTL on SSC17. Using the online Primer3 software, we designed 19 and three pairs of primers to amplify the full-length DNA and cDNA sequences of porcine *BMP2*, respectively. The primer sequences and the lengths of the corresponding amplified fragments are listed in Table S1 [see Additional file 1: Table S1]. To detect variants in the *BMP2* gene, we used the DNA of 10 DLY pigs with different genotypes (5 *AA*, 2 *AG* and 3 *GG* genotypes) at the 60 K GWAS tag SNP rs80965549. To sequence *BMP2* cDNA, total RNA was extracted from the articular cartilage tissue of 10 DLY pigs with 5 *AA* and 5 *AG* genotypes at the SNP rs80965549. Then, cDNA was synthesized from 1 µg of total RNA by using the PrimeScript™ RT Reagent kit (Takara). Polymerase chain reaction (PCR) was performed in a 25 µL reaction mixture containing 50 ng template DNA, 0.5 µL of each dNTP, 0.5 µL of each primer, 2.5 µL buffer, 1.5 µM MgCl_2_, and 0.5 µL of Tag DNA polymerase (Takara) and using a PTC-200 thermal cycler (Bio–Rad). The PCR products were sequenced on both strands by using a BigDye™ Terminator v3.1 kit (Applied Biosystems) on a 3130 DNA Analyzer (Applied Biosystems). The genomic sequences were assembled and analyzed for SNP detection using the SeqMan program in the DNASTAR software.

### Analysis of the cartilage transcriptome

Total RNA was isolated from cartilage tissue from eight DLY-P3 pigs. They represented two groups (4 animals per group) with extremely different CL phenotypes and different genotypes (*AA* and *GA*) at the GWAS tag SNP rs80965549. The purified mRNA was fragmented for sequencing on the MGISEQ-2000 platform at BGI Co. Ltd. The raw sequencing data of these samples averaged 10.92 G. Using the STAR software, clean reads were mapped to the *Sus scrofa* reference genome build 11.1. Gene expression levels were estimated using the StringTie software [35] for each pig. Differentially-expressed genes (DEG) with a fold change of ≥ 2 and a divergence probability of ≥ 0.8 between the two groups based on CL phenotype were identified using the DESeq2 software [36].

### Cell culture

A total of 10^6^ cells/ml of the murine osteoblast-like cell line MC3T3-E1 and of the osteocyte-like cell line MLO-Y4 were cultured in a 37 °C incubator with 5% CO_2_. Then, 500 μL of 0.25% trypsin were added to digest cells for 1 to 2 min at 37 °C and DMEM cell culture medium containing 10% fetal bovine serum (Invitrogen) was added to terminate the digestion and for subculture. Live cells were then cryopreserved in liquid nitrogen at − 196 °C.

### Electrophoretic mobility shift assay (EMSA)

Nuclear protein was extracted from the cultured cells (MC3T3-E1 and MLO-Y4) using the Nuclear Extraction Kit (Abcam) and protein concentration was measured with the BCA Protein Assay Kit (Abcam). The following two 31-bp biotin-labeled oligonucleotides were used as probes for the wild-type (Wt) and mutant (Mt) sequences, respectively: 5’-CAAGGTAAATCTCAACAATATTTTGCAGTTT-3’ (Wt) and 5’-CAAGGTAAATCTCAATAATATTTTGCAGTTT-3’ (Mt). The composition of the reaction system varied according to the experimental design: (1) for the negative control group: 1.5 μL 10 × binding buffer, 0.5 μL biotin-labeled probe, and 13 μL ddH_2_O to a final volume of 15 μL; (2) for the experimental group: 1.5 μL 10 × binding buffer, 5 μL nuclear extract, 1 μL (20 fmol) wild-type or mutant biotin-labeled probe, and 7.5 μL ddH_2_O to a final volume of 15 μL; and (3) for the competition assay group: 1.5 μL 10 × binding buffer, 5 μL nuclear extract, 1 µL (20 fmol) biotin-labeled probe, 1 μL (1–4 pmol) nonlabelled competitor probe, and 6.5 μL ddH_2_O to a final volume of 15 μL. The binding reaction was incubated at 20–25 °C for 30 min. After adding 1.5 μL of loading buffer, the DNA–protein complexes were separated by electrophoresis on a 5% polyacrylamide gel in 0.5 × TBE buffer at 10 V/cm for 1 to 1.5 h at 4 °C. The reaction samples were transferred to a nylon membrane by wet electroblotting at 390 mA for 60 min. Transferred DNA was crosslinked for 60 s under UV light (254 nm) at 120 mJ/cm^2^, and the protein-DNA complexes were detected by a chemical imaging system (iBright FL1000, Thermo Fisher Scientific).

### Luciferase reporter assay

To measure the effect of the putative causal SNP (rs320706814) upstream of the *BMP2* gene on transcriptional activity, both allelic forms (*TT* and *CC*) of a 201 bp fragment centered around the QTL were PCR-amplified from genomic DNA and subcloned into the Kpnl and BgIII sites of the pGL3-promoter vector (Promega). MC3T3-E1 and MLO-Y4 cells were grown to approximately 80% confluence. Cells were transiently co-transfected with the firefly luciferase reporter construct (100 ng) and a Renilla luciferase control vector (100 ng; Promega) using the Hieff TransTM kit (YEASEN) according to the manufacturer’s recommendations. Cells were incubated for 48 h before lysis. The luciferase activities were measured using the Dual-Luciferase Reporter Assay System (Promega). At least three independent experiments were performed in triplicate for each cell line. The activities of the mutant promoter were compared to those of the wild-type promoter based on normalized luciferase expression. Statistical analysis was performed with analysis of variance (ANOVA). A *P* value lower than 0.01 was considered as statistically significant.

## Results

### A genome-wide significant QTL for CL on SSC17

**Fig. 1:**
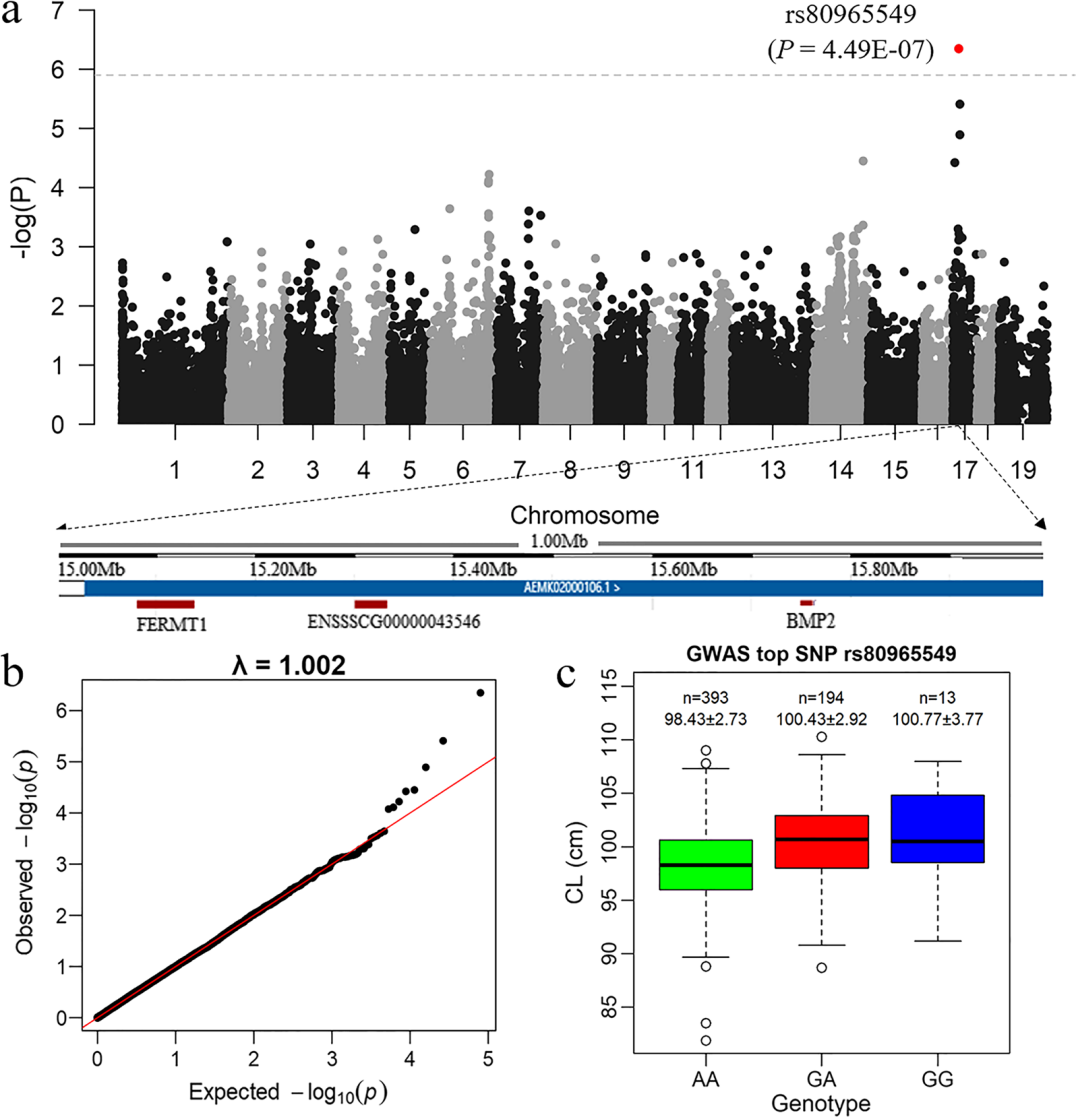
Genome-wide association results for carcass length in 686 DLY pigs. **a** Manhattan plot, with the genome-wide significance threshold at − log_10_P > 6 indicated by the dotted line. The top SNP rs80965549 is located in the intergenic region upstream of the *BMP2* gene. **b** Quantile–quantile (QQ) plot. The red line represents the 95% confidence level for the null hypothesis of no association between SNPs and the trait. The black dots represent the *P* values of all SNPs. **c** Effect of the top SNP rs80965549 on carcass length.

To identify QTL for body size in pigs, we performed a GWAS for CL using 60 K SNP genotype data from a discovery population of 686 DLY pigs (denoted as DLY-P1). The genomic inflation factor (λ) was equal to 1.0018 (Fig. 1b), which indicates that population stratification was properly adjusted for. GWAS detected one association signal with genome-wide significance at the SNP rs80965549 (*P* = 4.49E−07) on SSC17 (Fig. 1a). In the DLY-P1 population, carriers of the *G* allele of rs80965549 (*GG* and *GA*) had a 2 cm greater CL than noncarriers (Fig. 1c) and the frequency of the favorable *G* allele was only 0.18. The most significant SNP explained approximately 8% of the phenotypic variance in the DLY-P1 population. To verify this association signal, we analyzed the association between rs80965549 and CL in a replication population of 608 DLY pigs (denoted as DLY-P2) and also obtained a significant result (*P* = 2.52E−05).

By LD decay analysis, we found that the LD (r^2^) dropped below 0.2 at distances greater than 200 kb in the DLY-P1 population [see Additional file 2: Fig. S1]. Thus, the causal mutations and causal genes were expected to be located within a region from 500 kb upstream to 500 kb downstream of the detected GWAS signal. Notably, the 1 Mb region centered on the rs80965549 SNP contains only three annotated protein-coding genes, *FERM1*, *ENSSSCG00000043546*, and *BMP2*. The *FERM1* gene encodes the fermitin family member 1 protein, which is involved in integrin signaling and cell adhesion. Comparative genomic analysis of the *ENSSSCG00000043546* gene showed that it has no homologs in humans, dogs, cows, or mice, and its function is unknown. The *BMP2* gene encodes bone morphogenetic protein 2, a secreted ligand of the TGF-beta superfamily of proteins, which is known to play an important role in bone and cartilage development [37]. Therefore, among the three candidates, we considered *BMP2* to be the most likely candidate gene for the QTL on SSC17.

### No mutation in the *BMP2* gene accounted for the effect of the SSC17 QTL

Next, we determined whether there was any variant within the *BMP2* gene that could underlie the observed QTL effect. To this end, we sequenced the full-length DNA sequences of the *BMP2* gene of 10 DLY pigs with different rs80965549 genotypes (5 *AA*, 2 *GA* and 3 *GG*) and CL phenotypes (high versus low) and detected 46 variants in the *BMP2* gene [see Additional file 3: Table S2], of which 43 were in introns, two were synonymous variants, and one was a missense variant (rs45432445). We also sequenced full-length cDNA fragments of *BMP2* in 10 other DLY pigs (including 5 *AA* and 5 *GA* genotypes at SNP rs80965549), which revealed no splicing site mutation that might cause deletions or insertions in the *BMP2* transcript sequence [see Additional file 4: Table S3]. The SNP rs45432445 in *BMP2* causes a serine to leucine change at amino acid residue 257 (i.e., S257L). However, this missense mutation was predicted to be “neutral” by both the SIFT (score: − 2.29) and Polyphen-2 (score: 0.117) software. Moreover, the S257L mutation was not significantly associated with CL in DLY-P1 (*P* = 0.0778), which suggested that it was unlikely to be a causal mutation for the SSC17 QTL. Besides the S257L SNP, no variants in *BMP2* showed a genotypic segregation pattern similar to that of the GWAS tag SNP rs80965549 [see Additional file 3: Table S2], which implied that none of the other mutations detected in *BMP2* were likely related to the SSC17 QTL.

### The *BMP2* gene is differentially expressed in the cartilage of pigs with different QTL genotypes

After excluding the possibility of a causal mutation within the *BMP2* gene, we hypothesized that a regulatory variant that affects *BMP2* transcription might be responsible for the SSC17 QTL. Thus, to examine whether the expression level of *BMP2* differed between QTL genotypes, we compared the transcriptomic profiles in cartilage samples between *AA* and *GA* genotype groups (n = 4 per group) at the GWAS tag SNP rs80965549. The results showed that there were 486 differentially-expressed genes (DEG) between the two groups across the genome [see Additional file 5: Table S4]. Notably, the *BMP2* gene was identified as one of the DEG [see Additional file 6: Fig. S2a], while the other two positional candidates (*FERM1* and *ENSSSCG00000043546*) for the SSC17 QTL were not. The expression level of *BMP2* was nearly twofold lower in the heterozygote group than in the wild-type group. Furthermore, we used transcriptomic data from four heterozygotes and one wild-type homozygote for the SSC17 QTL, all of which were heterozygous for the SNP rs45434988 in an exon of *BMP2*, to detect allelic imbalances in *BMP2* transcripts. Based on the allelic expression ratio (AER) of the transcribed SNP rs45434988, we found that allelic expression imbalance (AER < 0.8 or AER > 1.2 [38]) occurred only in the four heterozygotes but not in the homozygote [see Additional file 6: Fig. S2b]. These results suggest that the QTL may influence CL by regulating the expression of *BMP2* during bone development.

### The QTL location was refined by using high-density SNP genotype data

**Fig. 2:**
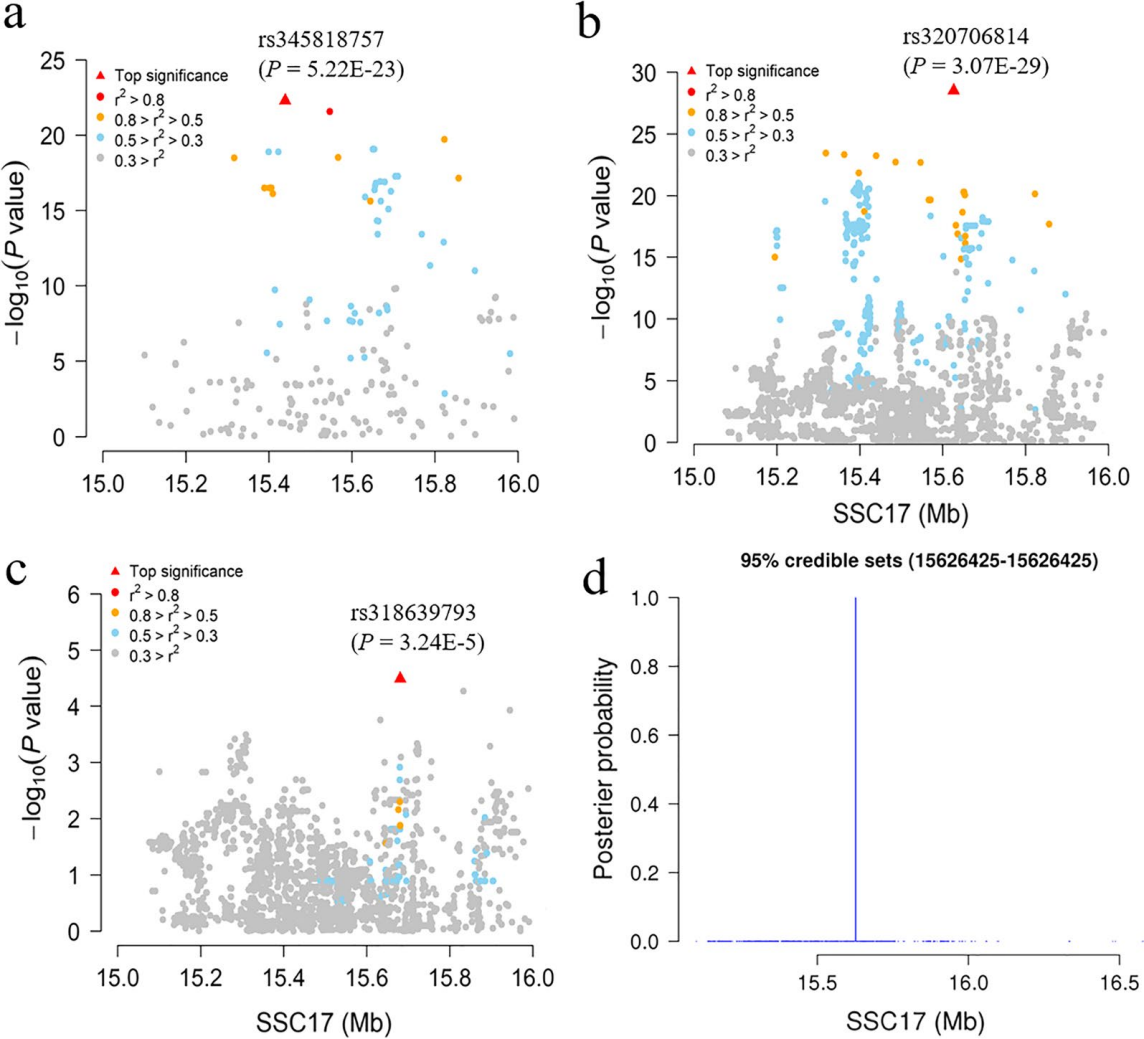
Fine-mapping of the SSC17 QTL for carcass length and detection of putative causal variants. **a** LocusZoom plot for the regional association analysis with an additional 196 SNPs that were genotyped and passed quality control. Different colors indicate different linkage disequilibrium values of the top SNP with other SNPs. **b** LocusZoom plot for the regional association analysis with imputed SNP genotypes. **c** LocusZoom plot for the association analysis conditioning on the lead SNP rs320706814, depicting the secondary association signal at SNP rs318639793. **d** Bayesian fine-mapping of the QTL. The 95% credible set of loci comprised only one variant (rs320706814), located at 15,626,425 bp, with the maximum posterior probability of causality

To fine-map the QTL on SSC17, we genotyped 267 additional SNPs within the candidate region from15 to 16 Mb and in the fine-mapping population DLY-Pf of 500 pigs that were selected from DLY-P1 and DLY-P2 based on high or low CL phenotype. No obvious problem of population stratification was observed in the selected individuals by PCA [see Additional file 7: Fig. S3]. Of the 267 SNPs, 196 passed the quality control thresholds. In the regional association analysis, SNP rs345818757, at 15,438,914 bp, was the most significant SNP (*P* = 5.22E−23) (Fig. 2a). Intriguingly, SNP rs345818757 was not in high LD (r^2^ > 0.8) with its neighboring SNPs, and no haplotype block contained rs345818757 [see Additional file 8: Fig. S4]. The three-LOD drop region of the QTL for CL, taken as its approximate confidence interval, ranged from 15.439 to 15.822 Mb.

### SNP rs320706814 was identified as a putative causal variant by imputation association and Bayesian analyses

To maximize the effectiveness of the association analysis, we imputed the genotypes of all SNPs within the QTL interval for the 500 pigs in the DLY-Pf population using a reference panel containing 561 re-sequenced individuals. In total, 9149 SNPs in the QTL interval met the quality control criteria and were included in the subsequent association analysis. The most significant SNP associated with CL was rs320706814 (*P* = 3.07E−29; at 15,626,425 bp), with a significance level that was five times higher than that of nearby SNPs (Fig. 2b). To assess the accuracy of the genotype imputation, SNP rs320706814 was genotyped for all animals in the DLY-Pf population using Sanger sequencing. Only 15 of the 500 individuals (3%) were not imputed correctly, suggesting that the imputation accuracy was acceptable. The association of SNP rs320706814 with CL slightly increased (*P* = 1.70E−29) after correcting the genotypes of these 15 pigs. In addition, the conditional association analysis of SNP rs320706814 indicated that SNP rs318639793 had an independent association with CL (*P* = 3.24E−05; at 15,680,821 bp) (Fig. 2c). This SNP was in very low LD with SNP rs320706814 (r^2^ = 0.082).

To determine the most likely causal variant underlying the QTL for CL, we genotyped the top 22 most significant SNPs that were detected in the DLY-Pf population by imputation-based association analysis for a total of 1501 pigs from the three DLY populations and examined their associations with CL phenotypes. Again, SNP rs320706814 showed the strongest association with CL (*P* = 2.01E−23), while the *P* values of the other 21 SNPs ranged from 1.82E−22 to 1.75E−10 [see Additional file 9: Table S5]. We also applied a Bayesian fine-mapping method to calculate a posterior probability of causality for variants in the candidate region. Results showed that the 95% credible set contained only one variant, i.e. the lead SNP rs320706814 (Fig. 2d). Thus, the statistical evidence prioritized rs320706814 as a potentially causal SNP for the CL association signal at this QTL.

**Fig. 3:**
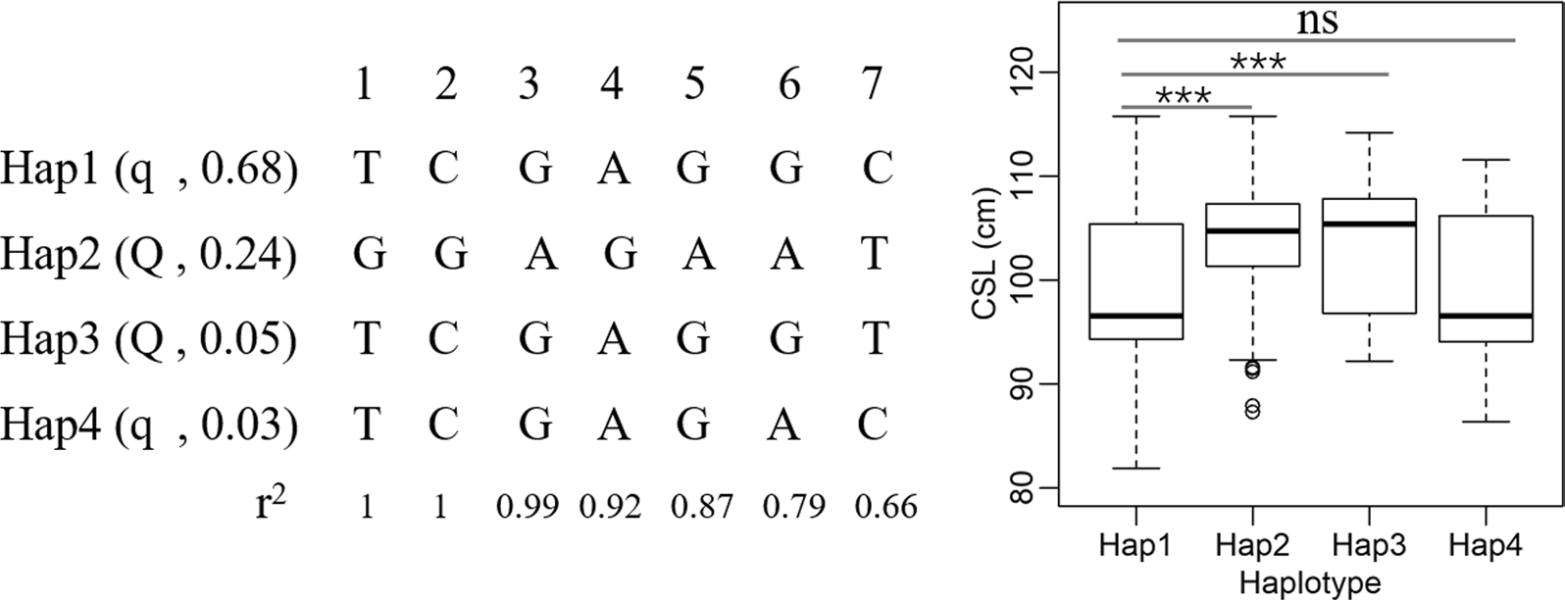
Comparison of the effects of four haplotypes on carcass length. The haplotypes are composed of the top seven SNPs that were most significantly associated with carcass length, specifically (in order from 1 to 7), rs342071386, rs333646524, rs345818757, rs323371124, rs336843722, rs343796635, and rs320706814. The linkage disequilibrium (r^2^) of the second-most significant SNP, #1, rs342071386, with other SNPs (including the most significant SNP, #7, rs320706814) are presented. Two effect-size categories (with favorable, *Q*, or unfavorable, *q*, effects on carcass length), with haplotype frequencies in parentheses

We also performed haplotype analysis in the DLY-Pf population, which was used for QTL fine-mapping. Haplotypes were constructed based on the top seven significant SNPs associated with CL. Four major haplotypes with frequencies higher than 3% were identified. By comparing the associations of these four haplotypes with CL, they could be divided into favorable and unfavorable haplotypes for CL, which were consistent with the segregation of alleles (*C*/*T*) at rs320706814 (Fig. 3). We noticed that haplotype 3, which had a favorable association with CL, was very similar in allele composition to haplotype 1, which had an unfavorable association with CL, except for the allele at the potential causal SNP rs320706814. This result not only explained why rs320706814 was in relatively low LD (r^2^ < 0.7) with other significant SNPs in the QTL interval but also confirmed that rs320706814 was likely a major contributor to the QTL.

### SNP rs320706814 affects carcass length by stimulating bone growth and segregates in multiple pig breeds

Across all DLY pigs, the *TT* genotype at SNP rs320706814 was estimated to increase CL by 4.39 cm compared with the *CC* genotype (Table 1). A significant association of the genotype at SNP rs320706814 with CL was also observed in 299 Yorkshire pigs (*P* = 9.2E−4), with a difference in CL between the *TT* and *CC* genotypes of 2.31 cm (Table 1).

**Table 1:** Effect of the top SNP rs320706814 on body length-related traits in three DLY pig cohorts and Yorkshire pigs

Cohorts	Traits	Genotypes						*P* value
		CC		CT		TT		
		N	Mean ± sd	N	Mean ± sd	N	Mean ± sd	
DLY-P1	CL (cm)	432	98.39 ± 3.78	252	100.16 ± 3.84	2	102.55 ± 3.18	2.54E−09
DLY-P2	CL (cm)	300	99.61 ± 5.58	288	102.98 ± 5.63	20	103.51 ± 5.19	1.54E−12
DLY-P3	CL (cm)	96	103.61 ± 4.57	110	106.35 ± 4.27	1	109.20	8.99E−06
	LTV_10th (cm)	95	2.94 ± 1.23	110	3.07 ± 0.19	1	3.00	4.95E−06
	LTV_mean (cm)	96	3.07 ± 0.16	110	3.15 ± 0.18	1	3.25	6.26E−04
	LTV_all (cm)	96	47.03 ± 2.94	110	48.10 ± 3.24	1	48.80	1.36E−02
	NTV	96	15.29 ± 0.60	110	15.28 ± 0.64	1	15.00	8.38E−01
	LLV_all (cm)	95	35.44 ± 5.18	110	36.21 ± 2.50	1	38.00	2.24E−02
DLY_all_	CL (cm)	828	99.44 ± 4.87	650	102.46 ± 5.25	23	103.67 ± 5.03	5.80E−24
Yorkshire	BL (cm)	208	79.29 ± 3.57	76	80.53 ± 3.33	15	81.60 ± 4.21	9.20E−04

*DLY-P1, DLY-P2, DLY-P3* the first, second and third cohorts of Duroc × (Landrace × Yorkshire) hybrid pigs

*DLY*_*all*_ the combination of all three DLY cohorts

*CL* carcass length, *BL* live body length; LTV_10th, LTV_mean, and LTV_all represent the length of the 10th thoracic vertebra, the average length of thoracic vertebrae and the total length of all thoracic vertebrae, respectively, *NTV* number of thoracic vertebrae, *LLV_all* total length of all lumbar vertebrae

*N* number of animals measured, *sd* standard deviation

Carcass length largely depends on the number of vertebrae and the length of each vertebra. In addition to CL, the number of thoracic vertebrae (NTV), the length of all thoracic vertebrae (LTV), and the length of the tenth thoracic vertebra (LTV10th) were also recorded in DLY-P3, which allowed us to estimate the effect of the SSC17 QTL on these traits. We found that SNP rs320706814 was significantly associated with CL (*P* = 2.16E−6), LTV10th (*P* = 3.13E−6), and LTV (*P* = 1.35E−2) but not with NTV (*P* = 0.83) (Table 1). These results indicate that the QTL increases CL by promoting longitudinal growth and the mineralization of each vertebra, rather than by increasing the number of vertebrae.

The frequency of the favorable allele *T* for CL at SNP rs320706814 was assessed in Western commercial pig breeds and Chinese indigenous pig breeds. The *T* allele was found to predominate in Landrace pigs at a frequency of 90.0% but it was less frequent in Duroc (9.4%) and Yorkshire (13.5%) pigs (Table 2), which resulted in a *T* allele frequency of approximately 23.2% in the DLY hybrids (Table 1). The rs320706814 SNP also segregated in wild boars and some Chinese native pig breeds, such as the Tibetan, Meishan, and Neijiang breeds, with frequencies of the *T* allele ranging from 6.7 to 27.9% (Table 2).

**Table 2:** Allele and genotype frequencies of SNP rs320706814 in pig breeds

Breeds	N	Genotypes			Frequency of the favorable allele *T*
		*TT*	*TC*	*CC*	
Chinese indigenous breeds					
Laiwu pig	14	0	0	14	0
Erhualian pig	29	0	0	29	0
Bamaxiang pig	12	0	0	12	0
Jinhua pig	10	0	0	10	0
Neijiang pig	8	0	1	7	0.067
Meishan pig	8	0	1	7	0.067
Tibet pig	59	1	19	39	0.178
Wild boar pig	68	9	20	39	0.279
Western commercial breeds					
Duroc	32	0	6	26	0.094
Landrace	35	29	5	1	0.900
Yorkshire	78	1	19	58	0.135

*N* number of individuals in each breed

### The rs320706814 SNP may weaken the enhancer and thereby reduce the expression of target genes

**Fig. 4:**
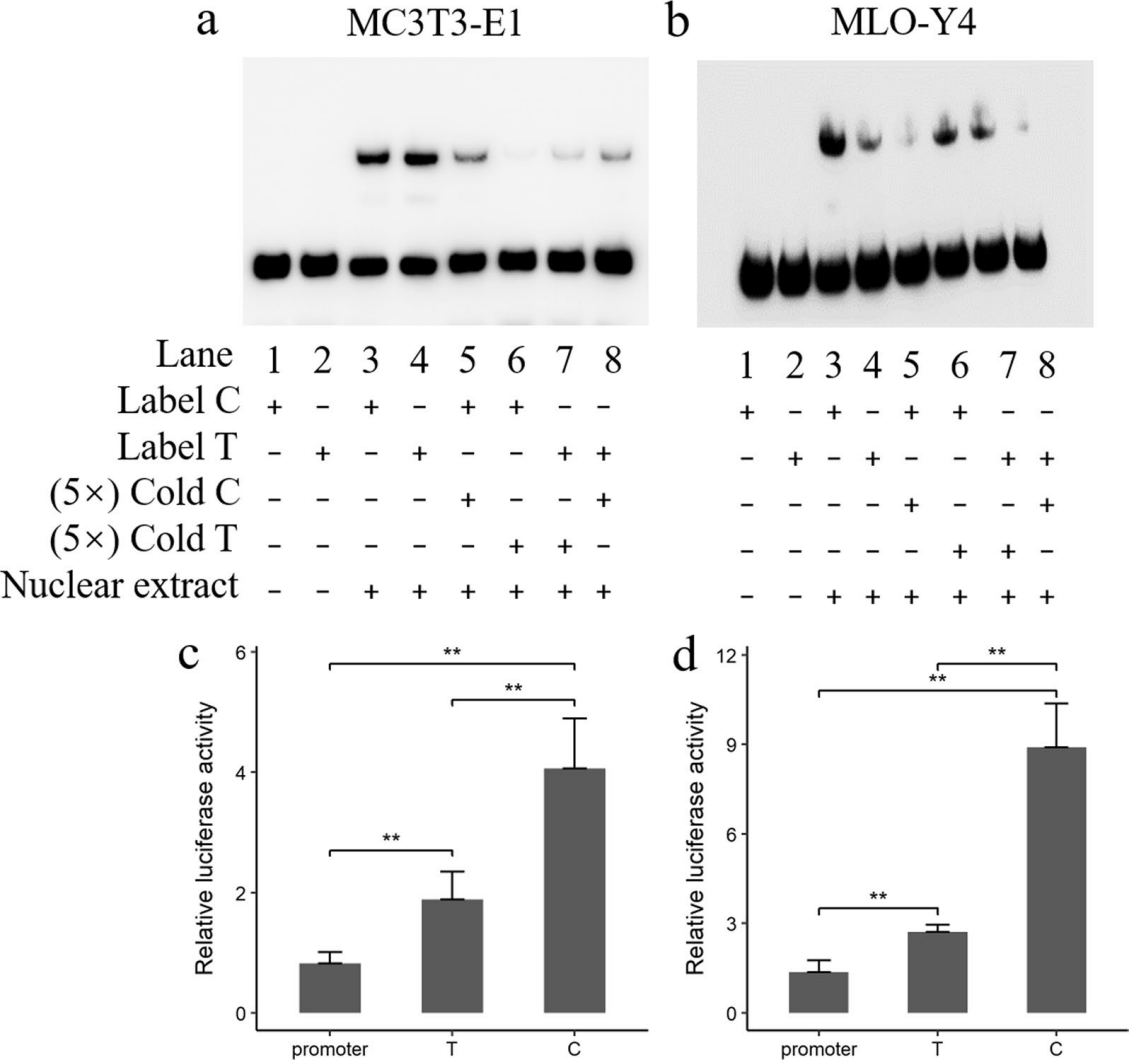
EMSA and transfection assays assessing the significance of the SNP rs320706814 (C > T substitution) on gene expression. **a**, **b** EMSA using nuclear extracts from MC3T3-E1 and MLO-Y4 cells. **c**, **d** Luciferase assays of the rs320706814 reporter constructs in MC3T3-E1 and MLO-Y4 cells. Three independent experiments were performed in two cell lines. Three replicates were performed for each experiment. The means ± s.d. and comparative significance of each group are shown

As the rs320706814 SNP was an intergenic variant located 123.4 kb upstream of the *BMP2* gene, we examined its potential to affect gene expression. In silico analysis using two TFBS prediction tools (TFBIND and Jaspar) showed that the rs320706814 SNP results in loss of the *SOX5* transcription factor binding site and creation of a *CDXA* binding site. Then, we performed EMSA to evaluate differences in the binding of nuclear proteins between alleles of the rs320706814 SNP (*C* > *T*) in nuclear extracts from MC3T3-E1 cells and MLO-Y4 cells. Results showed that this SNP increased the DNA–protein binding affinity in mouse embryonic osteoblast precursor (MC3T3-E1) cells (Fig. 4a) but decreased the DNA–protein binding affinity in murine long bone osteocyte-Y4 (MLO-Y4) cells (Fig. 4b). Next, we performed luciferase reporter assays using oligonucleotides representing the alleles of the rs320706814 SNP cloned into pGL3-promoter vectors. As shown in Fig. 4c, d, enhanced luciferase activities were detected (*P* < 0.01) in cells transfected with vectors containing the oligonucleotides, which suggest that the region surrounding SNP rs320706814 acts as an enhancer to stimulate gene expression. Moreover, in both MC3T3-E1 cells and MLO-Y4 cells, the mutant allele *T* at SNP rs320706814 caused a decrease in luciferase activity compared to the wild-type allele *C* (*P* < 0.05; Fig. 4c, d), which is consistent with our previous findings that the expression level of *BMP2* was lower in mutant carrier than in wild-type animals. Taken together, these results suggest that SNP rs320706814 is a cis-regulatory variant that affects the expression of the *BMP2* gene, which in turn modifies the CL phenotype.

## Discussion

The Duroc × (Landrace × Large White) three-way cross has been the most widely used hybrid in commercial pig production. Understanding the genetic basis of phenotypic variation in DLY pigs will help to improve the use of heterosis and the effectiveness of purebred selection. Our GWAS unveiled the genetic architecture underlying carcass length in DLY pigs for the first time and revealed a unique significant associated SNP (rs80965549) at approximately 15.4 Mb on SSC17. This SNP was estimated to increase CL by 2.34 cm and explain 8% of the phenotypic variation in the DLY-P1 population; thus, it represents a major QTL. This result is consistent with empirical evidence that there are some common mutations with moderate to large effects on body size in livestock, e.g., dogs and cattle [39]. In contrast, many loci contribute to the genetic variation in height in humans, and most effects are small, with the median effect of genome-wide significant SNPs being only 1.43 mm [40]. The SNP rs80965549 falls within the QTL regions for body/carcass length that were identified previously in several GWAS using a combined intercross and backcross between Hampshire and Landrace pigs [41], a Large White × Landrace cross population [42], and a Large White population [17]. This not only supports the accuracy of the localization of our QTL but also indicates that this QTL is common to multiple commercial pig breeds.

Like for most detected QTL, to date no in-depth analysis of the QTL on SSC17 for CL has been reported. Here, we applied a series of statistical methods to refine the associated region and identify putative causative variants. First, we genotyped dense markers along the QTL region in a group of 500 individuals that were selected for this purpose (DLY-Pf). Second, non-genotyped SNPs in the target region were imputed by combining the high-density genotyped SNP data and a sufficient amount of WGS data. Third, we used a Bayesian method to improve the precision of the QTL location and extract a limited number of candidate causal variants. Finally, among the candidate variants, the most likely causal variant was pinpointed through a meta-analysis of genetic associations based on 1501 individuals. The results show that rs320706814 is likely the causal SNP for the trait. However, rs320706814 did not contribute to the haplotype block identified for the QTL region in the DLY pigs, which can be explained either by the much smaller extent of LD in the DLY crossbreeds compared with their parent lines or by the fact that the rs320706814 SNP is likely an ancient mutation, since it is also present in the Chinese wild boar. It is worth noting that we have not completely ruled out the possibility that SNP rs320706814 is just a marker that is closely linked to the causal mutation. Other non-SNP mutations (e.g., indels or structural variants) were not included in this analysis, and within the 383-kb fine-mapped QTL region, the current genomic assembly may contain gaps that overlap with the actual causal mutation. Another possibility is that more than one causal mutation underlies the QTL and that the SNP rs320706814 is one of these. Indeed, the conditional analysis on rs320706814 revealed a secondary association signal in the QTL region. The mechanistic basis underlying this association needs further investigation.

Associations of SNPs that are adjacent to the *BMP2* locus with body size have been identified by GWAS in humans [43] and pigs. In stem cells, *BMP2* can induce chondrogenic differentiation, osteogenic differentiation, and endochondral ossification [37, 44]. The *BMP2* gene has been prioritized as a human growth-associated gene by a number of supporting studies and by DEPICT, which is a data-driven, integrative method that uses gene sets reconstituted on the basis of large-scale expression data to prioritize genes and gene sets [43]. By transcriptomic profile analysis of porcine cartilage tissues, we confirmed the relationship of genotype of the GWAS lead SNP rs80965549 with expression of the *BMP* gene. These results support the previous inference that *BMP2* underlies the QTL for body size.

It is known that many QTL are caused by mutations in regulatory elements. In addition, members of the BMP gene family, including *BMP2*, *BMP4*, *BMP5*, and *GDF6*, are known to be under the control of distant cis-regulatory elements [45]. Here, we provide further evidence that the most likely causal SNP rs320706814 identified by statistical analysis is a regulatory mutation located upstream of the *BMP2* gene. Luciferase assays in both MC3T3-E1 and MLO-Y4 cells consistently showed that this mutation resides in an enhancer but downregulates gene expression. However, EMSA showed that the rs320706814 SNP had opposite effects on DNA–protein binding affinity in the two cell types, which suggests the existence of different transcription factors (TF) that interact with the enhancer motif in different types of cells.

To the best of our knowledge, only two strong putative causal mutations have been identified for QTL for vertebrae number in pigs: the Pro192Leu missense mutation in the *NR6A1* gene [46] and the g.20311_20312ins291 insertion in the *VRTN* gene [47]. These two mutations are expected to affect body/carcass length by increasing vertebrae number [48]. For the QTL on SSC17, we found that the putative causative SNP rs320706814 was not associated with vertebrae number in DLY pigs but was associated with the length of individual thoracic vertebrae and the total length of all thoracic vertebrae. This is consistent with the effect of *BMP2* on bone growth.

The significance and efficiency of selecting a favorable QTL allele for a trait depends on its frequency in the population and on its effect on the phenotype. We found that the rs320706814 SNP (*C*/*T*) segregated in all three parental lines of the DLY cross and in some Chinese native breeds. The frequency of the favorable *T* allele for CL of the rs320706814 SNP was higher in Chinese wild boars than in any of the seven tested Chinese domestic pig breeds (Table 2), which may be attributable to genetic drift, inbreeding, or other factors. However, the frequency of the favorable allele at this SNP in European wild boar remains to be studied. Interestingly, the favorable *T* allele was almost fixed in the Landrace breed, with a frequency of 90%, while its frequency was only around 10% in the Duroc and Yorkshire breeds. Since the Duroc and Yorkshire breeds, similar to the Landrace breed, have undergone strong selection for CL, the low frequency of the favorable allele is unexpected. One explanation may be that this locus has a significant negative effect on other traits in Duroc and Yorkshire, although this seems unlikely given the high frequency of the favorable *T* allele in Landrace. Instead, it is more likely that the favorable allele at this locus was distributed at different frequencies in the progenitors of these three commercial breeds; a low initial frequency of a favorable allele in the progenitor population will limit the increase in its frequency. In short, under different genetic backgrounds, the effect of this locus on body length and other traits needs to be further evaluated to make better use of its value in breeding.

## Conclusions

This study elucidated the causality and molecular mechanism underlying a prominent GWAS signal near the *BMP2* locus on SSC17 that affects body size in Western commercial pig breeds. Our results indicate that the rs320706814 SNP is the main cause of the effect. This variant was found to alter the ability of the enhancer to regulate the transcription of the *BMP2* gene, which results in a change of approximately 4 cm in carcass length in DLY pigs. The favorable allele is found in both Western commercial breeds and Chinese native breeds. This information will contribute to the genetic improvement of body size in current breeding programs.

## Supplementary Information

**Additional file 1. Table S1. **Sequence information for primers used for cDNA and DNA sequencing of the *BMP2* gene.

**Additional file 2. Figure S1. **LD (r^2^) decay as a function of inter-SNP distance in the DLY-P1 population. LD decay is a fast and effective tool for linkage disequilibrium decay analysis based on variants. LD (r^2^) dropped below 0.2 at distances greater than 200 kb in the DLY-P1 population.

**Additional file 3. Table S2. **Screening for mutations in the *BMP2* gene in ten DLY pigs with different genotypes at the GWAS tag SNP rs80965549.

**Additional file 4. Table S3. **Mutations identified by sequencing the *BMP2* cDNA from ten DLY pigs with *AA* or *GA* genotypes at the GWAS tag SNP rs80965549.

**Additional file 5. Table S4. **List of differentially expressed genes identified in cartilage between different QTL genotypes (Qq vs. qq).

**Additional file 6. Figure S2. **Transcriptome analysis of cartilage tissue. **a** Volcano plot of differentially expressed genes (DEG). **b** Allelic expression ratio (AER) analysis with a transcribed SNP rs45434988 (G>A) in *BMP2* from four heterozygotes (Qq) and one wild-type homozygote (qq) for the SSC17 QTL. The five individuals were all heterozygous for rs45434988. The Y-axis represents the ratio of RNA-seq reads carrying different alleles of rs45434988 (G/A) in a sample. Allelic expression imbalance was determined by an AER greater than 1.2 or less than 0.8, indicated by dotted lines.

**Additional file 7. Figure S3. **Assessment of the population stratification of all DLY animals by PCA. The red dots represent DLY-P1, the green dots represent DLY-P2, and the blue dots represent DLY-P3.

**Additional file 8. Figure S4. **Haplotype block view of the region from 15.395 to 15.487 Mb. Haplotype structures viewed using Haploview software. The red mark was the top SNP rs345818757.

**Additional file 9. Table S5. **The associations between some candidate casual mutations and carcass length in all three DLY populations (N=1501).

## Data Availability

The datasets used and/or analyzed during the current studies are available from the corresponding author upon reasonable request.
