# Supplementary material for: Identification and validation of a regulatory mutation upstream of the BMP2 gene associated with carcass length in pigs
Source: Genet Sel Evol. 2021 Dec 14;53:94. doi: 10.1186/s12711-021-00689-0 (PMC8670072; doi:10.1186/s12711-021-00689-0)
Supplement: Supplementary file 1 — Additional file 1. Table S1. Sequence information for primers used for cDNA and DNA sequencing of the BMP2 gene. [file 12711_2021_689_MOESM1_ESM.docx]

**Table S1 Sequence information for primers used in cDNA and DNA sequencing of the *BMP2* gene**

| No. | Forward primer sequence (5’- 3’) | Reverse primer sequence (5’- 3’) | Size (bp) | Template |
| --- | --- | --- | --- | --- |
| 1 | GAGCGCCTCGTGGATTAC | GCTGGTGCTGTCCTTGTC | 639 | DNA |
| 2 | TAGCACTGAGCGACCGAC | AGCCCTAATAAAACACCAA | 729 | DNA |
| 3 | GGTGTTTTATTAGGGCTGTT | CCCATTCTCAATCGGTCT | 756 | DNA |
| 4 | GACCGATTGAGAATGGGATG | CTCACCTTCGTGGTGGAAG | 575 | DNA |
| 5 | CAGCGAGTTTGAGTTGCG | CTCCCACGAGGCTTAGAT | 770 | DNA |
| 6 | CCCTATCTAAGCCTCGTG | CTCCTTCCCATTATCAAC | 636 | DNA |
| 7 | ATGTCACAGCCAGGAGTT | ATCAGCATCAGCTAGGAA | 374 | DNA |
| 8 | TGTACGGTCCGTCATCAA | TTTCACCAATCACCCAGA | 764 | DNA |
| 9 | GTAAAGGGCTAAAGGACC | GCCTTTCATAATCACGCT | 748 | DNA |
| 10 | TGGCAGCGTGATTATGAAAG | ATTTTGTGGCTGCCTTTTTG | 665 | DNA |
| 11 | TTACACCTTGTCATACCG | GCTCTGATTTGAACCCTA | 585 | DNA |
| 12 | CTTCCGTGGCATATGGAAGT | CTGCAAACCATCACAACCAG | 609 | DNA |
| 13 | AAGGGTTATTTACCAAGA | GTGTATCAAAGAGGCATT | 750 | DNA |
| 14 | CATCCGCCCTCTTTACTTCA | AGCCATGCACACCCTAAAAC | 565 | DNA |
| 15 | TGGAAGATACTTCGGACC | CCCAAGCAAACAATAAAC | 861 | DNA |
| 16 | AGAAGGTCAACCCACATA | AAACTCAACATCCACGAT | 656 | DNA |
| 17 | TGCAAAATGTCATCGTGGAT | CGGTGGGGACAGAAGTTAAA | 630 | DNA |
| 18 | GGAAGAACTGCCAGAAAT | AGGTACAGCATGGAGATG | 768 | DNA |
| 19 | AATCACGCCATCGTCCAG | TTGCCAGAGTAACCTTCC | 618 | DNA |
| 20 | GAGCGCCTCGTGGATTAC | GCTGGTGCTGTCCTTGTC | 639 | DNA |
| 21 | AACAGAACTCAGTGCCATC | TTGCCAGAGTAACCTTCC | 551 | DNA |
| 22 | CTCCTCTTCCCAGCCCTCG | GCCTTTTGCCCTCATCTT | 535 | DNA |
| 23 | ACCGATTGAGAATGGGATG | TCAAACTCGCTGAGGACG | 385 | DNA |
| 24 | AGCACTGAGCGACCGACC | CCTGGGGAAGCAGCAACG | 544 | cDNA |
| 25 | AGGCGAAGGAAGGGAACC | GTCACGGGGAACTTGGAG | 691 | cDNA |
| 26 | GTAGCAGTTTCCATCACCG | CCACCTTTTCGTTCTCGT | 651 | cDNA |
