# Supplementary figures and images for: Identification and validation of a regulatory mutation upstream of the BMP2 gene associated with carcass length in pigs
Source: Genet Sel Evol. 2021 Dec 14;53:94. doi: 10.1186/s12711-021-00689-0 (PMC8670072; doi:10.1186/s12711-021-00689-0)

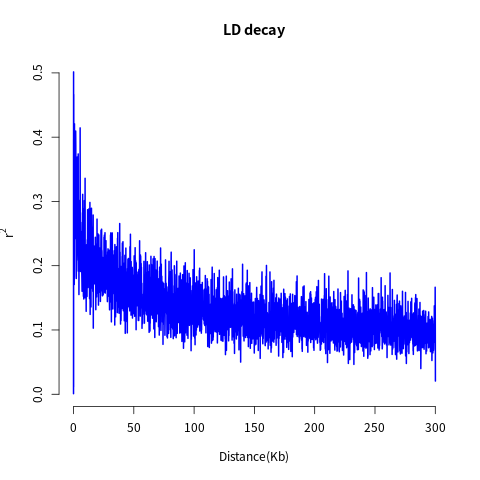

Supplement: Supplementary file 2 — Additional file 2. Figure S1. LD (r2) decay as a function of inter-SNP distance in the DLY-P1 population. LD decay is a fast and effective tool for linkage disequilibrium decay analysis based on variants. LD (r2) dropped below 0.2 at distances greater than 200 kb in the DLY-P1 population. [file 12711_2021_689_MOESM2_ESM.png]

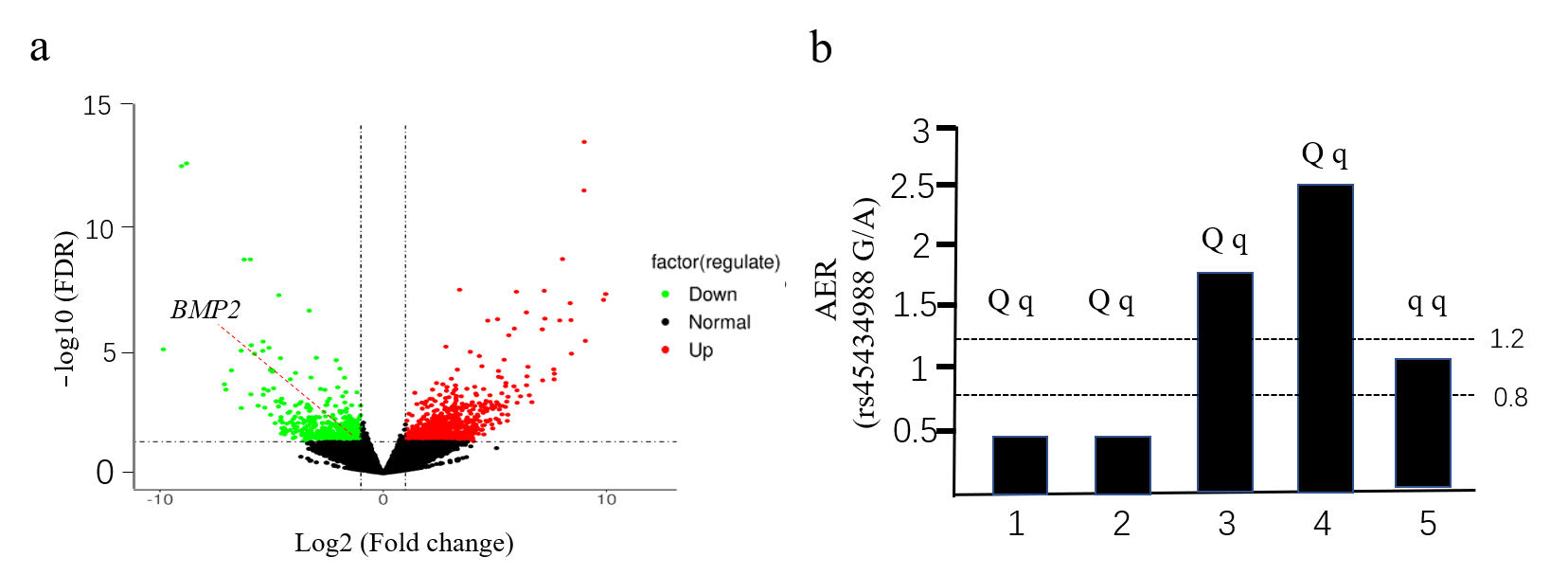

Supplement: Supplementary file 6 — Additional file 6. Figure S2. Transcriptome analysis of cartilage tissue. a Volcano plot of differentially expressed genes (DEG). b Allelic expression ratio (AER) analysis with a transcribed SNP rs45434988 (G>A) in BMP2 from four heterozygotes (Qq) and one wild-type homozygote (qq) for the SSC17 QTL. The five individuals were all heterozygous for rs45434988. The Y-axis represents the ratio of RNA-seq reads carrying different alleles of rs45434988 (G/A) in a sample. Allelic expression imbalance was determined by an AER greater than 1.2 or less than 0.8, indicated by dotted lines. [file 12711_2021_689_MOESM6_ESM.png]

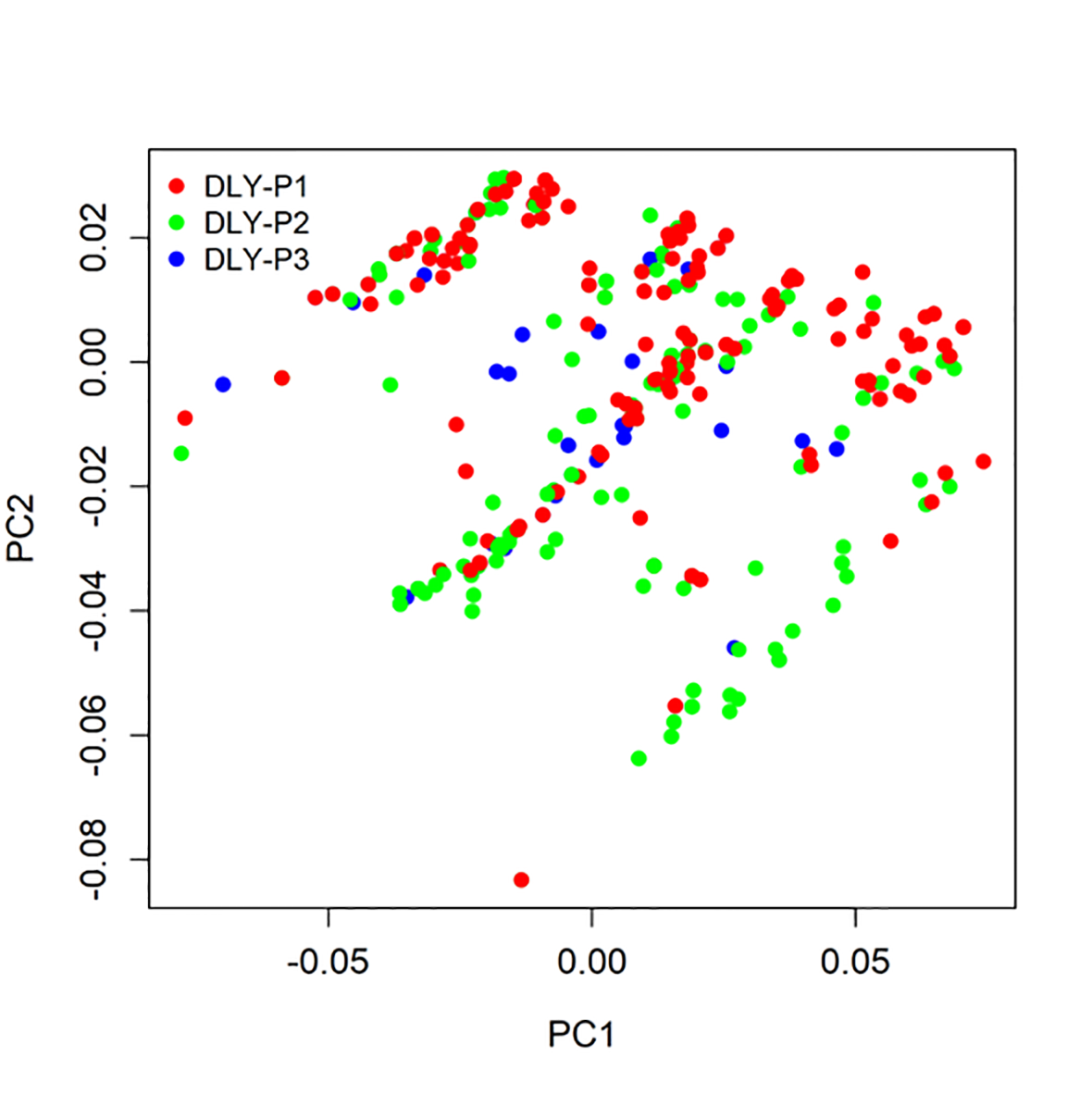

Supplement: Supplementary file 7 — Additional file 7. Figure S3. Assessment of the population stratification of all DLY animals by PCA. The red dots represent DLY-P1, the green dots represent DLY-P2, and the blue dots represent DLY-P3. [file 12711_2021_689_MOESM7_ESM.png]

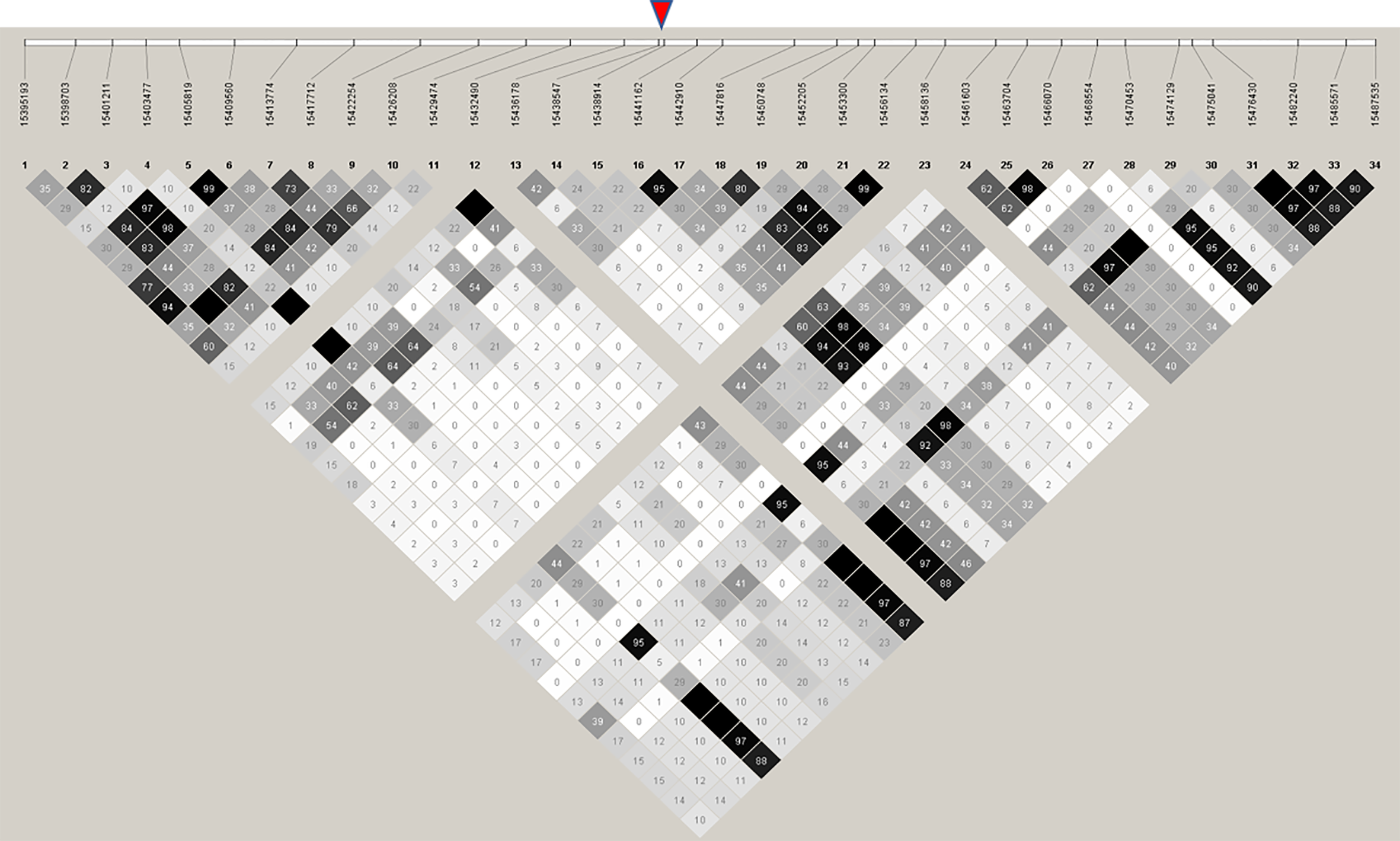

Supplement: Supplementary file 8 — Additional file 8. Figure S4. Haplotype block view of the region from 15.395 to 15.487 Mb. Haplotype structures viewed using Haploview software. The red mark was the top SNP rs345818757. [file 12711_2021_689_MOESM8_ESM.png]
