# Supplementary material for: Identification and validation of a regulatory mutation upstream of the BMP2 gene associated with carcass length in pigs
Source: Genet Sel Evol. 2021 Dec 14;53:94. doi: 10.1186/s12711-021-00689-0 (PMC8670072; doi:10.1186/s12711-021-00689-0)
Supplement: Supplementary file 3 — Additional file 3. Table S2. Screening for mutations in the BMP2 gene in ten DLY pigs with different genotypes at the GWAS tag SNP rs80965549. [file 12711_2021_689_MOESM3_ESM.docx]

**Table S2 Screening for mutations in the *BMP2* gene in ten DLY pigs with different genotypes at the GWAS tag SNP rs80965549**

| No. | variant ID | Chr: Position (bp) | No.1  (A,A^a^; 99.5cm^b^) | No.2  (A,A; 99.9cm) | No.3  (A,A; 88.8cm) | No.4  (A,A; 90cm) | No.5  (A,A; 90.3cm) | No.6  (G,A; 113.6cm) | No.7 (G,A; 113.5cm) | No.8 (G,G; 104.8cm) | No.9 (G,G; 108.3cm) | No.10  (G,G; 113.9cm) | consequence type |
| --- | --- | --- | --- | --- | --- | --- | --- | --- | --- | --- | --- | --- | --- |
| 1 | rs80791204 | 17:15750750 | G,C | G,C | G,G | G,C | G,G | G,G | G,G | G,G | G,G | G,G | exon (synonymous variant) |
| 2 | /^c^ | 17:15752947 | TGTTTGGA,TGGA | TGTTTGGA,TGGA | TGGA,TGGA | TGTTTGGA,TGGA | TGTTTGGA,TGGA | TGGA,TGGA | TGGA,TGGA | TGGA,TGGA | TGGA,TGGA | TGGA,TGGA | intron (indel) |
| 3 | rs1112602200 | 17:15752945 | T,C | T,C | T,T | T,C | T,C | T,T | T,T | T,T | T,T | T,T | intron |
| 4 | rs330422380 | 17:15753389 | G,A | G,A | G,G | G,A | G,G | G,G | G,G | G,G | G,G | G,G | intron |
| 5 | rs339451460 | 17:15753393 | A,T | A,T | A,A | A,T | A,A | A,A | A,A | A,A | A,A | A,A | intron |
| 6 | rs1112951525 | 17:15753462 | T,G | T,G | T,T | T,G | T,T | T,T | T,T | T,T | T,T | T,T | intron |
| 7 | rs1112334521 | 17:15753647 | T,C | T,C | T,T | T,C | T,C | T,T | T,T | T,T | T,T | T,T | intron |
| 8 | rs1113243583 | 17:15753814 | C,T | C,T | T,T | C,T | T,T | T,T | T,T | T,T | T,T | T,T | intron |
| 9 | / | 17:15754143 | T,T | T,G | T,T | T,G | T,G | T,T | T,T | T,G | T,T | T,G | intron |
| 10 | / | 17:15754148 | T,T | T,G | T,T | T,G | T,G | T,T | T,T | T,G | T,T | T,G | intron |
| 11 | / | 17:15754169 | A,A | A,G | A,A | A,G | A,A | A,A | A,A | A,G | A,A | A,G | intron |
| 12 | rs1110960618 | 17:15754237 | C,T | C,T | C,C | C,T | C,T | C,C | C,C | C,C | C,C | C,C | intron |
| 13 | rs1108547706 | 17:15754354 | C,C | C,C | C,C | C,C | C,T | C,C | C,C | C,C | C,C | C,C | intron |
| 14 | rs1110125886 | 17:15754439 | A,G | A,G | A,A | A,G | A,A | A,A | A,A | A,A | A,A | A,A | intron |
| 15 | / | 17:15754455 | A,G | A,G | A,A | A,G | A,A | A,A | A,A | A,A | A,A | A,G | intron |
| 16 | / | 17:15754456 | A,G | A,G | A,A | A,G | A,A | A,A | A,A | A,A | A,A | A,G | intron |
| 17 | rs1108686212 | 17:15754703 | A,G | A,G | A,A | A,G | A,G | A,A | A,A | A,A | A,A | A,A | intron |
| 18 | rs80851316 | 17:15755709 | C,T | C,T | T,T | ­ --^d^ | ­ -- | T,T | T,T | ­ ­-- | ­ ­-- | ­ ­-- | intron |
| 19 | rs80962837 | 17:15755711 | C,A | C,A | C,C | ­ ­-- | ­ -- | C,C | C,C | ­ ­-- | ­ ­-- | ­ -- | intron |
| 20 | rs1109179296 | 17:15756377 | T,C | T,C | T,T | T,C | T,T | T,T | T,T | T,T | T,T | T,T | intron |
| 21 | rs80818556 | 17:15756409 | A,G | A,G | A,A | A,G | A,A | A,A | A,A | A,A | A,A | A,A | intron |
| 22 | / | 17:15756501 | A,T | A,T | A,A | A,T | A,A | A,A | A,A | A,A | A,A | A,A | intron |
| 23 | / | 17:15756517 | A,T | A,T | A,A | A,T | A,A | A,A | A,A | A,A | A,A | A,A | intron |
| 24 | / | 17:15756527 | G,A | G,A | G,G | G,A | G,G | G,G | G,G | G,G | G,G | G,G | intron |
| 25 | rs1113292239 | 17:15756760 | T,C | T,C | T,T | T,C | T,T | ­ ­-- | ­ -- | T,T | T,T | T,T | intron |
| 26 | rs713245007 | 17:15756864 | T,G | T,G | T,T | T,G | T,T | ­ --­ | ­ --­ | T,T | T,T | T,T | intron |
| 27 | rs696122962 | 17:15756905 | C,T | C,T | ­ ­-- | ­ ­-- | ­ ­-- | ­ --­ | ­ ­-- | ­ ­-- | ­ ­-- | ­ ­-- | intron |
| 28 | rs702716971 | 17:15756906 | G,A | G,A | ­ ­-- | ­ ­-- | ­ ­-- | ­ --­ | ­ ­-- | ­ --­ | ­ ­-- | ­ ­-- | intron |
| 29 | rs712766139 | 17:15756990 | T,C | T,C | T,T | T,C | T,T | ­ ­-- | ­ ­-- | T,T | T,T | T,T | intron |
| 30 | rs1112260561 | 17:15757177 | G,A | G,A | G,G | G,A | G,G | ­ ­-- | ­ ­-- | G,G | G,G | G,G | intron |
| 31 | / | 17:15757876 | A,G | A,G | A,A | G,A | A,A | ­ ­-- | ­ --­ | A,A | A,A | A,A | intron |
| 32 | rs708707272 | 17:15757955 | G,A | G,A | G,G | G,A | A,A | ­ ­-- | ­ --­ | G,G | G,G | G,G | intron |
| 33 | rs1109280013 | 17:15758083 | T,G | T,G | T,T | T,G | G,G | ­ ­-- | ­ ­-- | T,T | T,T | T,T | intron |
| 34 | rs704016162 | 17:15758181 | G,A | G,A | G,G | G,A | G,G | ­ ­-- | ­ ­-- | G,G | G,G | G,G | intron |
| 35 | rs693104894 | 17:15758275 | T,G | T,G | T,T | T,G | T,T | ­ ­-- | ­ --­ | T,T | T,T | T,T | intron |
| 36 | rs702824202 | 17:15758356 | G,C | G,C | C,C | G,C | C,C | ­ ­-- | ­ ­-- | C,C | C,C | C,C | intron |
| 37 | rs701999113 | 17:15758570 | A,A | A,A | A,C | A,A | A,C | A,A | A,A | A,A | A,A | A,A | intron |
| 38 | rs697010486 | 17:15758772 | A,C | A,C | A,C | A,C | C,C | C,C | C,C | C,C | C,C | C,C | intron |
| 39 | rs706281844 | 17:15758777 | T,T | T,T | T,T | T,T | T,C | T,T | T,T | T,T | T,T | T,T | intron |
| 40 | / | 17:15758983 | AGTA,AGAAGTA | AGTA,AGAAGTA | AGTA,AGAAGTA | AGTA,AGAAGTA | AGTA,AGAAGTA | AGAAGTA,AGAAGTA | AGAAGTA,AGAAGTA | AGAAGTA,AGAAGTA | AGAAGTA,AGAAGTA | AGAAGTA,AGAAGTA | intron (indel) |
| 41 | rs1113716061 | 17:15759234 | C,T | C,C | C,C | ­ ­-- | C,T | C,C | C,C | C,C | C,C | C,C | intron |
| 42 | rs1112563973 | 17:15759328 | C,C | C,C | C,T | ­ ­-- | C,C | C,C | C,C | C,C | C,C | C,C | intron |
| 43 | / | 17:15759377 | TTATTCT,TTCT | TTATTCT,TTCT | TTATTCT,TTCT | ­ ­-- | TTATTCT,TTCT | TTCT,TTCT | TTCT,TTCT | TTCT,TTCT | TTCT,TTCT | TTCT,TTCT | intron (indel) |
| 44 | / | 17:15760431 | ­ ­-- | ­ ­-- | G,A | G,A | G,A | A,A | A,A | A,A | A,A | A,A | exon (synonymous variant) |
| 45 | rs45432445 | 17:15760739 | ­ ­-- | ­ ­-- | C,C | C,C | C,T | C,C | C,C | C,C | C,C | C,C | exon (missense variant) |
| 46 | / | 17:15771042 | A,A | A,A | A,A | A,A | A,T | A,A | A,A | A,A | A,A | A,A | intron |

^a^ Genotype of rs80965549.

^b^ Carcass length value of the animals.

^c^ The variant has no refSNP ID in Genbank.

^d^ The genotype was not determined.
