# Supplementary material for: Identification and validation of a regulatory mutation upstream of the BMP2 gene associated with carcass length in pigs
Source: Genet Sel Evol. 2021 Dec 14;53:94. doi: 10.1186/s12711-021-00689-0 (PMC8670072; doi:10.1186/s12711-021-00689-0)
Supplement: Supplementary file 4 — Additional file 4. Table S3. Mutations identified by sequencing the BMP2 cDNA from ten DLY pigs with AA or GA genotypes at the GWAS tag SNP rs80965549. [file 12711_2021_689_MOESM4_ESM.docx]

**Table S3 Mutations identified by sequencing of the *BMP2* cDNA from ten DLY pigs with AA or GA genotypes at the GWAS tag SNP rs80965549**

| No. | variant ID | Chr: Position (bp) | No.1  (A, A^a^; 100.5cm^b^) | No.2  (A, A; 100.4cm) | No.3  (A,A; 99.9cm) | No.4  (A,A; 99.5cm) | No.5  (A,A; 100.4cm) | No.6  (G, A; 111.9cm) | No.7  (G, A; 113.6cm) | No.8  (G, A; 109.5cm) | No.9  (G, A; 113.5cm) | No.10  (G, A; 108.8cm) |
| --- | --- | --- | --- | --- | --- | --- | --- | --- | --- | --- | --- | --- |
| 1 | rs80791204 | 17:15750750 | G,C | G,G | G,C | G,C | G,C | G,G | G,G | G,G | G,G | G,G |
| 2 | /^c^ | 17:15760431 | G,G | G,A | G,A | G,A | G,A | A,A | A,A | A,A | A,A | A,A |
| 3 | rs45432445 | 17:15760739 | C,T | C,T | C,C | ­ ­--^d^ | C,C | C,C | C,C | C,C | C,C | C,C |

^a^ Genotype of rs80965549.

^b^ Carcass length value of the animals.

^c^ The variant has no refSNP ID in Genbank.

^d^ The genotype was not determined.
