# Supplementary material for: Identification and validation of a regulatory mutation upstream of the BMP2 gene associated with carcass length in pigs
Source: Genet Sel Evol. 2021 Dec 14;53:94. doi: 10.1186/s12711-021-00689-0 (PMC8670072; doi:10.1186/s12711-021-00689-0)
Supplement: Supplementary file 9 — Additional file 9. Table S5. The associations between some candidate casual mutations and carcass length in all three DLY populations (N=1501). [file 12711_2021_689_MOESM9_ESM.docx]

**Table S5 The associations between some candidate casual mutations and carcass length in all three DLY populations (N = 1501)**

| SNP ID | variant | Chr: Position (bp) | *P*-value |
| --- | --- | --- | --- |
| [rs320706814](http://asia.ensembl.org/Sus_scrofa/Variation/Explore?db=core;r=17:15626415-15626435;source=dbSNP;v=rs320706814;vdb=variation;vf=2876733) | G/T | 17: 15626425 | 2.01E-23 |
| [rs345818757](http://asia.ensembl.org/Sus_scrofa/Variation/Explore?db=core;r=17:15438904-15438924;source=dbSNP;v=rs345818757;vdb=variation;vf=27513862) | G/A | 17: 15438914 | 1.82E-22 |
| [rs342071386](http://asia.ensembl.org/Sus_scrofa/Variation/Explore?db=core;r=17:15318056-15318076;source=dbSNP;v=rs342071386;vdb=variation;vf=23837381) | G/A | 17: 15318066 | 1.78E-21 |
| [rs333646524](http://asia.ensembl.org/Sus_scrofa/Variation/Explore?db=core;r=17:15362348-15362368;source=dbSNP;v=rs333646524;vdb=variation;vf=15571563) | G/A | 17: 15362358 | 2.91E-21 |
| [rs336843722](http://asia.ensembl.org/Sus_scrofa/Variation/Explore?db=core;r=17:15546290-15546310;source=dbSNP;v=rs336843722;vdb=variation;vf=18708746) | G/A | 17: 15546300 | 4.08E-21 |
| [rs323371124](http://asia.ensembl.org/Sus_scrofa/Variation/Explore?db=core;r=17:15486482-15486502;source=dbSNP;v=rs323371124;vdb=variation;vf=5490691) | A/C | 17: 15486492 | 3.70E-19 |
| [rs333233735](http://asia.ensembl.org/Sus_scrofa/Variation/Explore?db=core;r=17:15822366-15822386;source=dbSNP;v=rs333233735;vdb=variation;vf=15166681) | A/G | 17: 15822376 | 4.85E-18 |
| [rs345314946](http://asia.ensembl.org/Sus_scrofa/Variation/Explore?db=core;r=17:15710321-15710341;source=dbSNP;v=rs345314946;vdb=variation;vf=27019577) | T/C | 17: 15710331 | 6.23E-17 |
| [rs342754935](http://asia.ensembl.org/Sus_scrofa/Variation/Explore?db=core;r=17:15668216-15668236;source=dbSNP;v=rs342754935;vdb=variation;vf=24507936) | G/C | 17: 15668226 | 6.59E-17 |
| [rs318768659](http://asia.ensembl.org/Sus_scrofa/Variation/Explore?db=core;r=17:15704542-15704562;source=dbSNP;v=rs318768659;vdb=variation;vf=975363) | G/A | 17: 15704552 | 1.32E-16 |
| [rs339716958](http://asia.ensembl.org/Sus_scrofa/Variation/Explore?db=core;r=17:15662021-15662041;source=dbSNP;v=rs339716958;vdb=variation;vf=21527452) | G/A | 17: 15662031 | 5.62E-16 |
| [rs337188278](http://asia.ensembl.org/Sus_scrofa/Variation/Explore?db=core;r=17:15316626-15316646;source=dbSNP;v=rs337188278;vdb=variation;vf=19046702) | T/G | 17: 15316636 | 8.49E-16 |
| [rs343796635](http://asia.ensembl.org/Sus_scrofa/Variation/Explore?db=core;r=17:15566851-15566871;source=dbSNP;v=rs343796635;vdb=variation;vf=25529874) | G/A | 17: 15566861 | 1.62E-15 |
| [rs331256680](http://asia.ensembl.org/Sus_scrofa/Variation/Explore?db=core;r=17:15650192-15650212;source=dbSNP;v=rs331256680;vdb=variation;vf=13227003) | T/C | 17:15650202 | 5.55E-15 |
| [rs324399380](http://asia.ensembl.org/Sus_scrofa/Variation/Explore?db=core;r=17:15653550-15653570;source=dbSNP;v=rs324399380;vdb=variation;vf=6499517) | T/C | 17:15653560 | 9.10E-15 |
| [rs345025103](http://asia.ensembl.org/Sus_scrofa/Variation/Explore?db=core;r=17:15650136-15650156;source=dbSNP;v=rs345025103;vdb=variation;vf=26735184) | G/A | 17:15650146 | 1.58E-14 |
| [rs318248308](http://asia.ensembl.org/Sus_scrofa/Variation/Explore?db=core;r=17:15650479-15650499;source=dbSNP;v=rs318248308;vdb=variation;vf=464827) | T./C | 17:15650489 | 1.58E-14 |
| [rs338232981](http://asia.ensembl.org/Sus_scrofa/Variation/Explore?db=core;r=17:15422244-15422264;source=dbSNP;v=rs338232981;vdb=variation;vf=20071744) | G/A | 17:15422254 | 4.60E-13 |
| [rs322343711](http://asia.ensembl.org/Sus_scrofa/Variation/Explore?db=core;r=17:15396960-15396980;source=dbSNP;v=rs322343711;vdb=variation;vf=4482719) | T/C | 17:15396970 | 5.35E-13 |
| [rs343909666](http://asia.ensembl.org/Sus_scrofa/Variation/Explore?db=core;r=17:15398693-15398713;source=dbSNP;v=rs343909666;vdb=variation;vf=25640786) | G/A | 17:15398703 | 6.08E-13 |
| [rs80965549](http://asia.ensembl.org/Sus_scrofa/Variation/Explore?db=core;r=17:15401201-15401221;source=dbSNP;v=rs80965549;vdb=variation;vf=165007) | G/C | 17:15401211 | 4.63E-11 |
| [rs320849773](http://asia.ensembl.org/Sus_scrofa/Variation/Explore?db=core;r=17:15821121-15821141;source=dbSNP;v=rs320849773;vdb=variation;vf=3016930) | G/A | 17:15821131 | 1.75E-10 |
